# Pattern recognition receptors in the development of nonalcoholic fatty liver disease and progression to hepatocellular carcinoma: An emerging therapeutic strategy

**DOI:** 10.3389/fendo.2023.1145392

**Published:** 2023-03-20

**Authors:** Chen Huang, Youlian Zhou, Jiemin Cheng, Xue Guo, Diwen Shou, Ying Quan, Hanqing Chen, Huiting Chen, Yongjian Zhou

**Affiliations:** ^1^Department of Gastroenterology and Hepatology, Guangzhou Key Laboratory of Digestive Diseases, Guangzhou Digestive Disease Center, Guangzhou First People’s Hospital, School of Medicine, South China University of Technology, Guangzhou, China; ^2^Department of Gastroenterology and Hepatology, the Second Affiliated Hospital, School of Medicine, South China University of Technology, Guangzhou, China

**Keywords:** NAFLD, HCC, PRR, innate immune signaling pathway, inflammation

## Abstract

Nonalcoholic fatty liver disease (NAFLD) is characterized by excessive lipid accumulation and has become the leading chronic liver disease worldwide. NAFLD is viewed as the hepatic manifestation of metabolic syndrome, ranging from simple steatosis and nonalcoholic steatohepatitis (NASH) to advanced fibrosis, eventually leading to cirrhosis and hepatocellular carcinoma (HCC). The pathogenesis of NAFLD progression is still not clear. Pattern recognition receptor (PRR)-mediated innate immune responses play a critical role in the initiation of NAFLD and the progression of NAFLD-related HCC. Toll-like receptors (TLRs) and the cyclic GMP-AMP (cGAMP) synthase (cGAS) are the two major PRRs in hepatocytes and resident innate immune cells in the liver. Increasing evidence indicates that the overactivation of TLRs and the cGAS signaling pathways may contribute to the development of liver disorders, including NAFLD progression. However, induction of PRRs is critical for the release of type I interferons (IFN-I) and the maturation of dendritic cells (DCs), which prime systemic antitumor immunity in HCC therapy. In this review, we will summarize the emerging evidence regarding the molecular mechanisms of TLRs and cGAS in the development of NAFLD and HCC. The dysfunction of PRR-mediated innate immune response is a critical determinant of NAFLD pathology; targeting and selectively inhibiting TLRs and cGAS signaling provides therapeutic potential for treating NALF-associated diseases in humans.

## 1 Introduction

Nonalcoholic fatty liver disease (NAFLD), which is newly defined metabolic associated fatty liver disease (MAFLD) is characterized by abnormal hepatic lipid accumulation and inflammatory syndrome, in addition to one of the following three criteria: overweight/obesity, type 2 diabetes mellitus (T2DM), or evidence of metabolic dysregulation (1–4). Recently, NAFLD is emerging as the rising public health problem that endangers human health worldwide (5, 6). The meta-analysis reveals that, the global prevalence of NAFLD has been approximately 25.2%, with the highest prevalence in Southeast Asia (42.0%) and the lowest rates reported from Africa (13.5%) (7, 8). The rising rates of obesity, insulin resistance, type 2 diabetes, and hyperlipidaemia contribute to the development of NAFLD (9). Thus, the prevalence of NAFLD is more than 70% among obese and diabetic patients (10). The hepatic metabolic disorder leads to chronic inflammation and compensatory tissue repair, which result in steatohepatitis with or without fibrosis. Current clinical studies indicated that around 10-20% of NAFLD patients would gradually develop into nonalcoholic steatohepatitis (NASH). NASH is characterized by liver inflammation, hepatocellular injury, and different degrees of hepatic fibrosis, which is mediated by innate immune responses. There are up to one-third of NASH cases might progress to advanced fibrosis or cirrhosis, then eventually develop into hepatocellular carcinoma (HCC) (11–14). The current mechanism studies indicate that lipotoxicity, inflammation, oxidative stress, mitochondrial dysfunction, and gut-liver axis are associated with the development of NAFLD to NASH and HCC (15–19). However, the pathogenesis of NAFLD progression is still not clear, and there are still no FDA-approved effective therapies available for end-stage NAFLD except liver transplantation (20, 21).

Growing evidence suggests that hepatic innate immune response plays an important role in triggering NAFLD initiation and progression. Although the innate immune system is considered to be a metabolic sensor against metabolic-related stresses, an overactive innate immune response becomes pathological. This scenario occurs in the development from NAFLD to NASH (22, 23). Pattern recognition receptors (PRRs) constitute the first line of defense in the host immune system, and are mainly expressed in the cell membrane, endosome, or cytoplasm of innate immune cells, such as macrophages, Dendritic cells (DCs), neutrophils, natural killer (NK) cells, and even in hepatocytes (24–26). During the initial event of the innate immune response, PRRs detect the evolutionarily conserved structures on invaded bacteria or viruses from cell death and tissue damage by pathogen-associated molecular patterns (PAMPs) or damage-associated molecular patterns (DAMPs), which lead to the activation of multiple intracellular signaling pathways and trigger the production of inflammatory cytokines and chemokines (24, 27, 28). Toll-like receptors (TLRs), a well-known family of endocytic PRRs, are widely expressed on all types of cells in the liver, including Kupffer cells (29, 30), hepatocytes (29, 31), hepatic stellate cells (HSCs) (32, 33) and biliary epithelial cells (29) (**Table 1**). cGAS is a newly discovered DNA sensor, which is considered to involve in the development of diverse liver diseases, such as viral hepatitis, NAFLD, drug-induced liver injury (DILI), and HCC (35, 43, 44). In this Review, we will summarize the currently available information regarding the role of PRRs on the pathogenesis and progression of NAFLD.

**Table 1:** The expression and action of TLRs and cGAS-STING in a variety of liver cells.

Liver cells	Detected PRRs expression	PRR function in the development of NAFLD and HCC
Kupffer cells	TLR2,3,4,7 cGAS-STING	1. TLR4 promotes the development of steatohepatitis-related HCC (34) 2. In NAFLD, Liver macrophages activates HSCs through cGAS-STING pathway (35) 3. MtDNA from hepatocytes of HFD-fed mice induced TNF-α and IL-6 expression in Kupffer cells through STING pathway (36) 4. Activation of TLR7 signaling in Kupffer cells induces hepatocyte death and inhibit Treg cells activities, leading to the progression of NASH (37)
Hepatocytes	TLR1-9, cGAS-STING	1. TLR4 or TLR9 deficiency improve liver fibrosis in Tak1Δhep mice (38) 2. In HCC, DNA damage induces cGAS-STING signaling in malignant hepatocytes (39)
hepatic stellate cells (HSCs)	TLR2,3,4	1. Radiotherapy activates HSCs through TLR4 signaling pathway and increases the potential of HCC metastasis (40) 2. Interfering TLR4 expression in HSCs inhibited the HSCs activation and attenuated the liver fibrosis (41)
biliary epithelial cells (BECs)	TLR1-9	1. Activation of TLRs in BECs triggers the production of cytokines or chemokines and anti-microbial peptides (42)

## 2 Toll-like receptors in NAFLD and HCC

**Figure 1:**
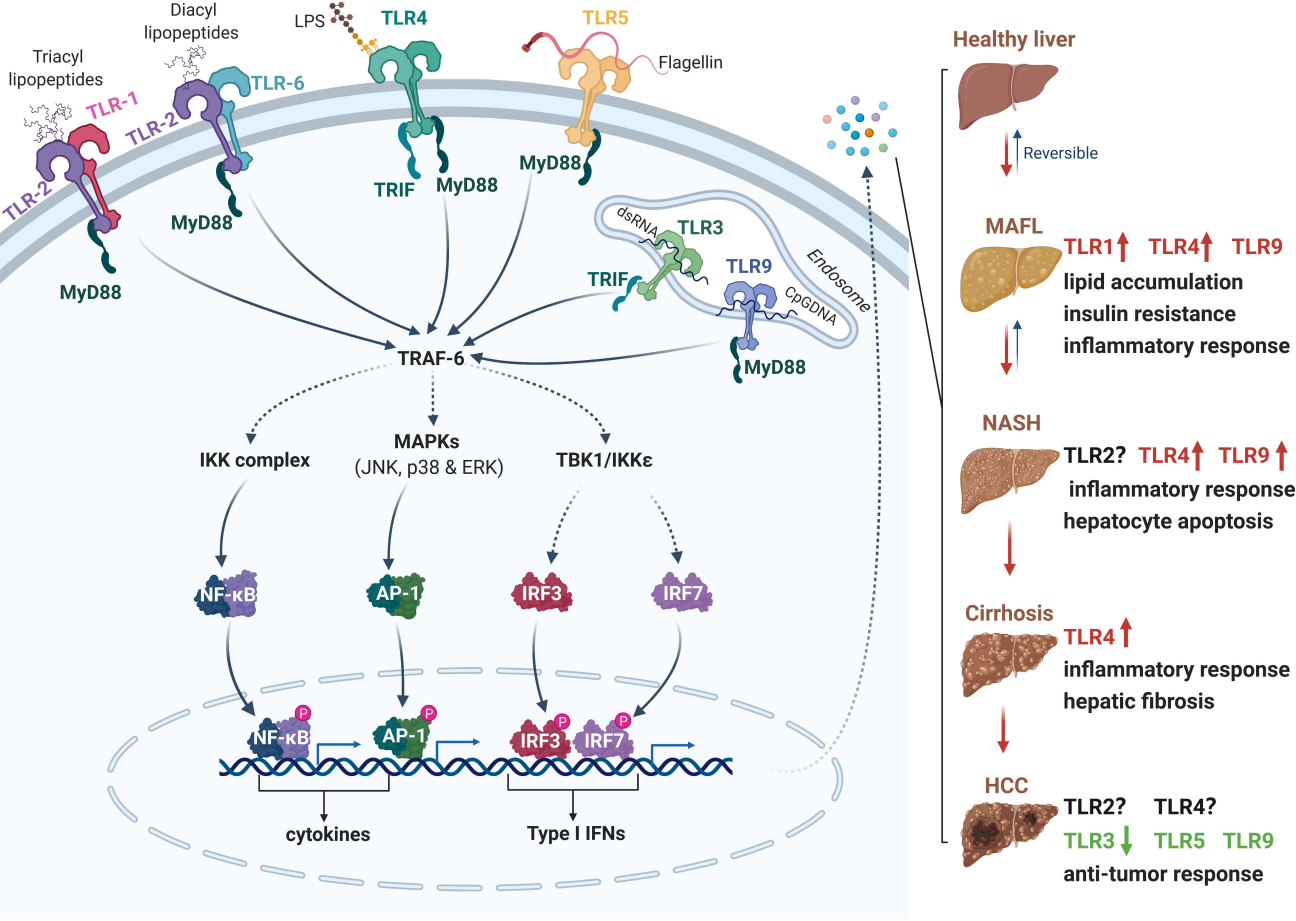
The role of Toll-like receptors (TLRs) in the pathogenesis of NAFLD and HCC. Following excessive high-fat diet intake and liver injury, PAMPs (e.g., LPS, flagellin, lipopeptides and DNA) from microbes and DAMPs released from damaged or dying cells are accumulated in the liver. TLRs recognize PAMPs and DAMPs and initiate downstream cascades in TRIF- or MyD88-dependent manner. The activation of IKK, MAPKs and TBK1 kinases trigger dimerization and translocation of NF-κB, AP-1 and IRF3/7 respectively, then induce the expression of several cytokines, chemokines, type I interferons, and other pro-apoptotic genes. The secretion of these active factors may contribute to insulin resistance, lipid accumulation, inflammation, hepatocyte apoptosis or anti-cancer responses. Different TLRs play diverse roles in the process of NAFLD to HCC. PAMPs, pathogen-associated molecular patterns; DAMPs, damage-associated molecular patterns; TRIF, TIR-domain-containing adapter-inducing interferon β; MyD88, myeloid differentiation primary response 88; IKK, IκB kinase; MAPK, mitogen-activated protein kinase; TBK1, TANK-binding kinase 1; NF- κB, nuclear factor kappa B; AP-1, activator protein 1; IRF, interferon regulatory factor. Red, accelerative effect; Green, protective effect.

TLRs are one of the earliest discovered PRRs, which form the cornerstone of in innate immune responses. To data, ten and twelve known functional TLRs have been identified and characterized in humans (TLR1-10) and twelve (TLR1-9 and 11-13) in mice, respectively. Some TLRs, such as TLR1, 2, 4, 5, 6 and 10, are found on the plasma membrane in the form of homodimers or heterodimers, which mainly recognize the membrane components of pathogenic microorganisms, including LPS, lipid A, lipoproteins, flagellin. The other TLRs including TLR3, 7, 8, and 9 are expressed on the intracellular membrane to recognize the microbial nucleic acid ligands. After binding with its ligand, the PRRs are activated and then recruit the downstream adaptors to elicit the assembly of signaling complexes by initiating the signaling cascades in MyD88-dependent or TRIF-dependent manner. The transcriptional factors, such as IRF3/IRF7, NF-κB, and AP-1, are activated and then translocated into the nucleus, resulting in the production of inflammatory cytokines, chemokines, and type I interferons. Recent accumulating evidence has demonstrated the association between NAFLD progression and TLR signaling pathway activation, particularly TLR4, TLR2 and TLR9 (**Figure 1**).

### 2.1 The role of TLRs in the pathogenesis of NAFLD/NASH

The mRNA levels of TLR 1-10 were determined in the liver samples of 11 patients with NAFLD and 11 health persons (45). The mRNA expression of TLR 1 to 5 was significantly higher in NAFLD patients, and the mRNA level of TLR 6 to 10 mRNAs was comparable between NAFLD patients and healthy controls. Another study found that the protein level of TLR6, but not TLR2, was gradually increased in the hepatocytes from the cohorts of NAFLD and NASH patients compared to those from normal liver biopsy, indicating that TLR6 contributed to inflammatory responses in the development of NASH (46). However, the effect of TLR2 on different models of NASH seems to be controversial. TLR2 deficient mouse exhibited severe hepatocellular damage and accelerated MCD diet-induced steatohepatitis and fibrosis (47). However, TLR2 deficiency has been shown to decrease the infiltration of inflammatory cells and the activation of inflammasome in choline-deficient amino acid-defined (CDAA) diet induced NASH model (48), indicating the critical role of TLR2 in the initiation of liver inflammation. TLR1 deficiency displayed significantly protective effect from the development of diet-induced NAFLD when compared to that in wild-type mice. Moreover, TLR1 expression was positively correlated with the level of *Holdemanella* genus, and was negative associated with the content of *Gemmiger* and *Ruminococcus* genera in NAFLD patients (49). Thus, TLR1 may be acted as a potential target in the treatment of NAFLD patients along with TLR1 high expression.

TLR4, a receptor of LPS, is widely expressed on the surface of Kupffer cells and hepatocytes. In the progression of NAFLD, metabolic alterations were usually accompanied by the increased intestinal permeability and gut microbiota dysbiosis (50, 51), which resulted in the elevating levels of circulating LPS in NAFLD/NASH patients and HFD or MCD-induced animal models (52, 53). The level of free fatty acids (FFAs), such as palmitic acid and stearic acid, was higher in NAFLD patients, indicating the association with the activation of TLR4 activation (54, 55). Moreover, the mRNA and protein levels of TLR4 were increased in liver samples from patients with hepatitis and cirrhosis (56). Overactivation of TLR4 increased the phosphorylation of NF-κB, MAPK and IRF3, and promoted the production pro-inflammatory cytokines in MyD88-dependent manner, which triggered severe liver injury and promoted the development of NAFLD. Genetic ablation of TLR4 resulted in a marked attenuation of liver inflammation in preclinical NASH models (57). In addition, absence of TLR4 downstream molecules IKKϵ and TBK1, or treatment with an IKKε- and TBK1-specific inhibitors showed a protective effect against hepatic steatosis (58, 59). These data demonstrate that TLR4 signaling promotes NAFLD progression, and inhibition of LPS release from the intestinal microbiota or usage of TLR4 signaling antagonism may be a feasible strategy for the prevention or treatment of NAFLD/NASH.

Similar with TLR4, the mRNA level of TLR9 was significantly upregulated in patients with NASH, but not in patients with hepatic steatosis (60). As a CpG DNA sensor, TLR9 could be activated by circulating mitochondrial DNA (mtDNA), which exhibited higher level in plasma from mice and patients with NASH (61–63). Pharmacological inhibition of TLR9 prevented the hepatic histopathological injury, proinflammatory cytokine production, and plasma ALT activity in NASH models (61). Furthermore, TLR9 deficiency significantly reduced liver weights and ameliorated hepatic steatosis in HFD-induced NASH mice, indicating the protective role of TLR9 in hepatic dysmetabolism (64).

### 2.2 The role of TLRs in the development of HCC

Approximately 80% of HCCs are developed by chronic liver disease, hepatic fibrosis and cirrhosis (65). Liver inflammation is considered as a risk factor to induce the development of HCC. TLRs has been reported to be a critical point in the pathogenesis and progression of HCC. Lin et al. reported that TLR4 was functionally expressed on two human hepatoma cell lines HepG2 and H7402. The activation of TLR4 induced by LPS significantly enhances COX-2/PGE_2_/STAT3 positive feedback loop and then triggers the proliferation of hepatoma cells and the occurrence of multidrug resistance in HCC chemotherapy (66). Unlike the higher expression of TLR4 in hepatitis and cirrhosis, the expression of TLR4 in liver samples of patients with hepatocarcinoma does not change (56). However, the level of TLR4 is higher in liver samples from relapsed HCC patients, indicating that TLR4 expression is a potential prognostic biomarker in HCC therapy (67, 68). On the contrary, TLR3 expression is associated with longer survival in HCC patients. Activation of TLR3 signaling by poly(I:C) increased intra-tumoral chemokine expression, NK-cell activation, and proliferation of tumor-infiltrating T and NK cells, which contribute to increasing cell death and decreased tumor growth. The effect of TLR3 on HCC is further confirmed by Bonnin et al. The level of TLR3 protein is downregulated in tumoral liver samples and exhibited much lower in 6 human HCC cells, including Hep3B, HepG2, and HuH7. Furthermore, the growth of tumors is accelerated in TLR3 deficiency HCC mice. Mechanistically, the absence of TLR3 prevents cell apoptosis and enhances tumor progression (69). Therefore, TLR3 and TLR4 may be potential prognostic biomarkers for HCC therapy in humans. Two SNPs in the exon of TLR2 were identified in HCC and were demonstrated to be associated with the risk of HCC in a single center-based case-control study (70). TLR2 is expressed in tumor-associated macrophages (TAM) of HCC and contributes to autophagy in the regulation of M2 macrophage polarization by high-mobility group box 1 (HMGB1)/NADPH oxidase 2 (NOX2) axis (71). TLR9 overactivation can enhance the therapeutical efficacy of PD-1 or PD-L1 on HCC treatment by promoting PD-L1 transcription and enhancing the phosphorylation of STAT3 Tyr705 (72). Recent research reported that TLR5 was an independent prognostic marker in HCC therapy, which function is similar to p53 (73). Pretreatment with TLR5 agonist effectively protects from LPS- and TNF-induced toxicity in liver and lung, and reudces the mortality, indicating that TLR5 agonist might be used as an adjuvant to enable the safe systemic application of TNF as an anticancer therapy (74).

Although TLRs have been demonstrated to be associated with chronic inflammation in the development of NAFLD/NASH, the effects of TLR signaling pathway on the progression of NAFLD-associated HCC remains unclear. TLR2 polarizes the TAMs to a pro-tumor M2 phenotype in HCC cells, and promotes the proliferation of HCC (71). However, another study performed revealed that knockdown TLR2 or downstream molecule MyD88 in B76/Huh7 cells promotes its proliferation and inhibit the apoptosis to increase the progression of hepatocarcinogenesis (75). In addition, TLR4 and TLR9 deficiency significantly reduces liver injury and blocks the progression of liver inflammation to hepatic fibrosis and HCC in a hepatic deletion of transforming growth factor-β-activated kinase 1 (Tak1ΔHep) mouse model (38), indicating that TLR3-mediated apoptosis may be a promising therapeutic target in HCC treatment (69). Taken together, further studies need to be investigated for the molecular mechanisms and therapeutic potential of TLRs in the development of HCC.

## 3 The cGAS-STING signaling axis in NAFLD and HCC

**Figure 2:**
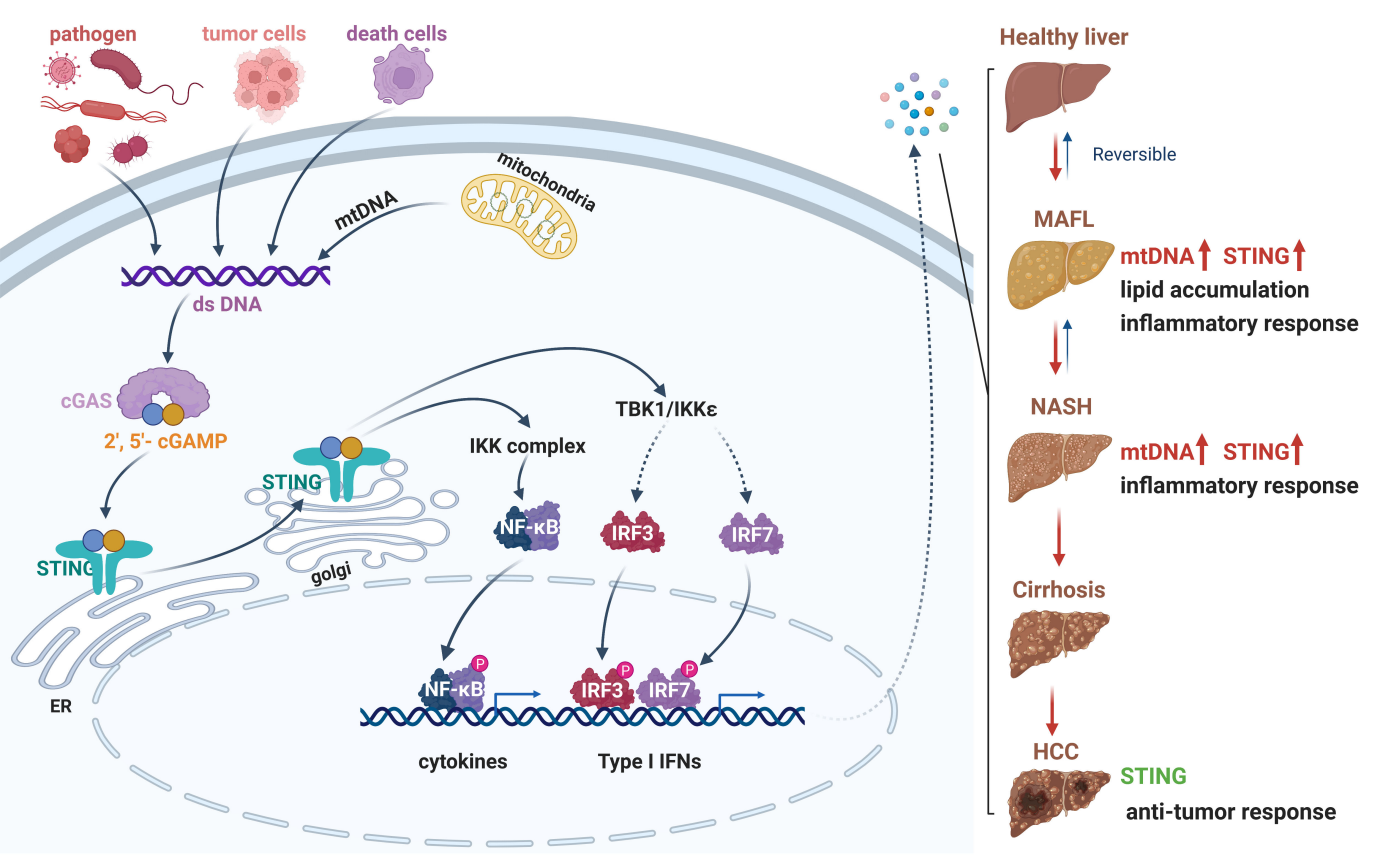
The cGAS-STING signaling pathway in the progression from NAFLD to HCC. A schematic detailing cytosolic dsDNA, which occurs through pathogen infection, tumor cells, death cells or cellular stress, can be recognized by cGAS. Enzymatic activation of cGAS results in the synthesis of 2′-3′ cGAMP. The binding of cGAMP enables STING translocation from ER membrane to Golgi, then recruits TBK1 and IKK complex. The phosphorylation of IRF3 and NF-κB by TBK1 and IKK enable these transcription factor dimerization and translocation to the nucleus to induce gene expression of several cytokines, type I interferons, and chemokines. Activation of cGAS-STING signaling axis could promote lipid accumulation, inflammation, or anti-cancer responses in different stage of liver disease. dsDNA, double-stranded DNA; cGAS, cyclic GMP-AMP synthase; 2′-3′ cGAMP, 2′3′ cyclic GMP-AMP; STING, stimulator of interferon genes; ER, endoplasmic reticulum; TBK1, TANK-binding kinase 1; IKK, IκB kinase; IRF3, interferon regulatory factor 3. Red, accelerative effect; Green, protective effect.

The cGAS-STING axis contains the cytosolic DNA sensor cGAS, the second messenger cyclic GMP-AMP (cGAMP) and cGAMP receptor stimulator of interferon genes (STING) (76–79). STING is expressed in non-parenchymal cells, including innate immune cells and hepatic stellate cells, and exerts an important role to maintain liver homeostasis (80). The cGAS-STING signaling is activated by pathogenic DNA from DNA virus or intracellular bacteria, and endogenous DNA, including mitochondrial and nuclear DNA in the cytosol in response to mitochondria stress, radiation therapy or autoimmune disorders (81–85). cGAS-STING axis is involved in the activation of IFN-I and pro-inflammatory responses against microbial infections *via* TBK1/IRF3 and NF-κB signaling, and STING- induced autophagy and lysosome-dependent cell death (86). Recently, cGAS-STING signaling has been demonstrated to be associated with various diseases, including inflammation, autoimmune diseases, metabolic disorders, and tumors (87–89). Here, we will highlight the pathogenical and therapeutical role of cGAS-STING axis in NAFLD to HCC (**Figure 2**).

### 3.1 NAFLD/NASH

In patients with NAFLD/NASH, STING has been demonstrated to be expressed and upregulated in myeloid cells (35). The level of STING protein in hepatic non-parenchymal cells including KCs and endothelial cells from patients with NAFLD is increased compared to the healthy controls. Increased numbers of STING positive monocyte-derived macrophages (CCR2^+^, S100A9^+^), Kupffer cells (CD68^+^) and CD163^+^ macrophages are found in liver samples from NASH patients with fibrosis (90). Consistent with NAFLD patients, the expression of STING and its downstream transcriptional factor IRF3 are significantly increased in the liver of HFD-fed mice (35, 91). The phosphorylation of JNK p46 and nuclear factor κB (NF-κB) p65 are enhanced in the liver of NAFLD mice, and promotes the production of tumor necrosis factor α (TNFα), interleukin (IL)1β, and IL6. However, the phosphorylation of transcriptional factors and activation of inflammatory cytokines are significantly lower in mice with STING disruption (STING^gt^). Moreover, the similar inflammatory status in myeloid cells was also found in mice with STING deficiency, indicating that myeloid-derived STING contributes to HFD-induced hepatic inflammatory response (35). Moreover, STING is deficiency in human and murine hepatocytes and the expression of STING is highly expressed in hepatic nonparenchymal cells (80, 92). However, the mechanism of HFD-triggered the activation cGAS-STING signaling is still not clear. It has been reported an abnormal liver mitochondrial function and high level of plasma mtDNA in NASH patients and HFD-induced NASH mice (61). Furthermore, STING deficiency attenuates the mtDNA-mediated TNF-α and IL-6 expression in KCs *in vitro (* 36). Thus, cGAS-STING signaling is activated by cytoplasmic mtDNA, and then initiates the excessive inflammatory response in the liver to aggravate the liver injury in NASH patients and mice.

Indeed, inhibition of STING or reduction of its downstream signaling pathway can attenuate the NAFLD/NASH symptoms. STING deficiency reduced inflammation, steatosis and fibrosis in livers in both methionine- and choline-deficient diet (MCD)- and HFD-induced murine models of NASH (36, 93). Knockdown STING or IRF3 alleviates the hepatic lipid accumulation, liver inflammation and hepatocyte apoptosis (91, 94). Furthermore, mice with STING disruption in myeloid cells exhibited less severe NASH symptoms (35). In addition, Remdesivir (RDV, GS-5734), an antagonist of STING, considerably restrains lipid accumulation, hepatic disorder and liver inflammation in HFD-fed mice (95). Therefore, targeting cGAS-STING axis may be an effectively therapeutic strategy for NAFLD/NASH in humans.

### 3.2 HCC

Activation of cGAS-STING axis aggravates the NAFLD symptom and accelerates the development of NASH. Interestingly, cGAS-STING play an opposite role in HCC progression. Thomsen et al. reported that STING-deficient mice displayed higher number of large tumors than wild type mice at late stages of HCC. Furthermore, activation of STING by treatment with a cyclic dinucleotide (CDN), a traditional STING agonist, could efficiently reduce HCC tumor size (44). In addition, *Lactobacillus rhamnosus* GG (LGG) treatment enhanced the efficacy of immune checkpoint blockade (ICB) therapies through inducing cGAS/STING-dependent IFN-β production in colorectal cancer therapy, which suggesting that cGAS-STING axis play a vital role in anti-tumor immune responses against HCC (96).

Recently, several studies have reported that the cGAS-STING signaling is a prognostic biomarker in HCC, and can be acted as an adjuvant to enhance the anti-tumor efficacy (97–99). cGAS/STING signaling were found to be related to the stages of HCC patients. The mRNA level of STING and its downstream target molecule including IFI16, STAT6 and NLRC3 is positive associated with the overall survival of HCC patients (97), indicating the cGAS-STING signaling factors can be used as potential prognostic biomarkers and therapeutical targets for HCC treatment in humans. Increasing evidence has been demonstrated the role of cGAS-STING signaling pathway to enhance the efficacy of radiotherapy and immunotherapy (100). Radiotherapy (RT) can induce DNA damage and lead to the accumulation of cytosolic DNA, which initiate the activation of cGAS-STING signaling and increase the RT-induced antitumor immunological therapy. Besides, the radiotherapy effect was enhanced by introducing exogenous cGAMP, or combination with cGAS-STING agonists and immunotherapy (101). Wehbe et al. developed a polymer some nanoplatform encapsulated with STING (STING-NPs) to enhance cGAMP delivery in the tumor and increase the half-life of cGAMP with 40-fold. In a B16-F10 melanoma tumor model, STING-NPs increase the response rates to αPD-L1 antibodies, and lead to increase the survival time in mice (102). Furthermore, STING-NP-treated tumor-bearing mice resulted in >50% and 80% reduction in tumor burden by cGAS-STING signaling in melanoma and breast adenocarcinoma models. E7766, a macrocycle-bridged stimulator of interferon genes (STING) agonist, was demonstrated its potent antitumor activity by prolonging the immune memory response in a liver metastatic tumor mouse model (103). Conversely, STING signaling has been reported to promote the tumorigenesis and progression in some cases. Upregulation of IFNβ induced by cGAS-STING promotes the expression of immune checkpoint molecules, such as cytotoxic T-lymphocyte-associated protein 4 (CTLA-4) and programmed cell death ligand 1 (PD-L1), which result in the inhibition of T-cell activation and immune evasion (104, 105). Therefore, targeting and selectively activating cGAS-STING signaling may have therapeutic potential for treating HCC in humans.

Lastly, there are no approved therapies for NAFLD and NASH. Given that the TLR4, TLR9 and cGAS-STING signaling pathway is activated under obesity conditions in NAFLD patients and mouse models, it is tempting to speculate that TLRs and cGAS-STING contribute to increased inflammation in the progression of NAFLD to NASH. In fact, some small-molecule inhibitors have been developed to target TLRs, cGAS-STING and their downstream components over the past several years. TAK-242, a specific TLR4 inhibitor, has been used for the treatment of rheumatoid arthritis (RA) (106). RU.521 and RU.365 are found to inhibit cGAS by binding at the active site of cGAS (107). Remdesivir, an antagonist of STING, is also considered to be a candidate for the treatment of NAFLD (95). On the contrary, the PRRs play an important roles in anti-tumor immunity. The two STING agonists, DMXAA (5,6-dimethylxanthenone 4-acetic acid) and E7766, were identified as molecules that exerted potent anti-tumor activity. Thus, it is important to pay attention to the administration time of antagonists or agonists that regulate the TLRs and cGAS-STING signaling pathway in order to prevent an increased risk of HCC. In conclusion, NAFLD has emerged as one of the most prevalent form of chronic liver disease worldwide due to overnutrition, genetic predisposition, gut-liver axis and immune disorder. PRRs and their downstream signaling closely contribute to NAFLD pathogenesis and accelerate the development of NASH, hepatic fibrosis, cirrhosis, and NAFLD-related HCC. In this review, we provide the comprehensive understanding of the molecular mechanism of PRRs to control liver inflammation in physiological and pathophysiological stages of NAFLD and HCC, and highlight the therapeutic targeting PRRs for the treatment of NAFLD/NASH and HCC.

## Author contributions

CH and YLZ conceptualized the topic of this review. JC, XG, DS, and YQ retrieved the literature and assisted in manuscript preparation. YJZ and HTC provided scholarly guidance on the topic and assisted in manuscript preparation. CH drafted the manuscript, HQC revised the manuscript. All authors have read and approved the final manuscript.
